# Unexpected finding of splenic peliosis in a traumatic spleen in a patient with cleidocranial dysplasia

**DOI:** 10.4102/sajr.v22i1.1371

**Published:** 2018-09-27

**Authors:** Prema Mohandas, Ahmed O.A. Krim, Paul Samson

**Affiliations:** 1 Department of General Surgery, Southland Hospital, New Zealand; 2 Department of Radiology, Southland Hospital, New Zealand

## Abstract

We present a case of traumatic rupture of the spleen in a man with cleidocranial dysplasia. The computed tomography imaging showed multiple low-grade lacerations of the spleen which initially led to conservative patient management. However, with clinical deterioration, the patient underwent an emergent splenectomy. Post-operative histology revealed splenic peliosis with multiple lacerations. The radiological and surgical management of post-traumatic splenic peliosis may differ from those with an otherwise normal spleen.

## Introduction

Splenic peliosis is an extremely rare benign condition characterised by the presence of blood-filled cysts varying in size from one millimetre to several centimeters^1^. While peliosis may occur in any organ belonging to the mononuclear phagocytic system (which includes the liver, spleen, bone marrow and lymph nodes), isolated splenic peliosis remains rare^1^. There have been no published cases of patients with splenic peliosis associated with cleidocranial dysplasia. To our knowledge, only 21 cases of isolated splenic peliosis have been reported in the literature^2^. Radiological imaging can be non-specific in splenic peliosis, and the most common differential diagnoses include lymphoma, vascular tumours or infective pathology^3^.

Cleidocranial dysplasia, also known as cleidocranial dysostosis, is a congenital disorder caused by a genetic mutation. It is a rare generalized skeletal dysplasia with a bone developmental abnormality. The condition is characterised by widened sutures, multiple wormian bones, hypoplasia or aplasia of the clavicles, abnormal teeth, vertebral and pubic symphysis abnormalities^4^.

## Case presentation

**FIGURE 1:**
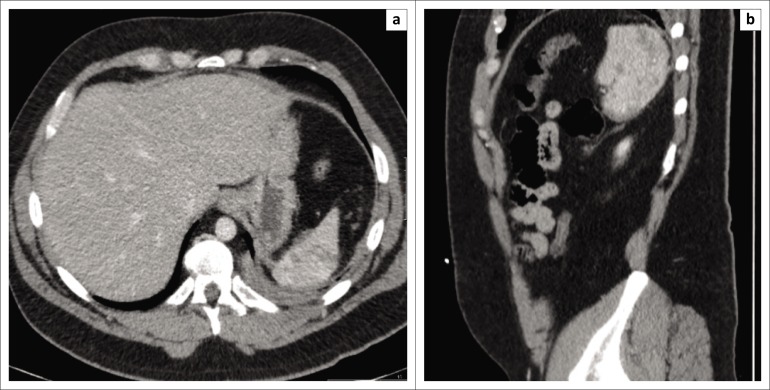
(a) Axial and (b) coronal images on the day of admission, showing multiple lacerations of the spleen with a subcapsular haematoma.

**FIGURE 2:**
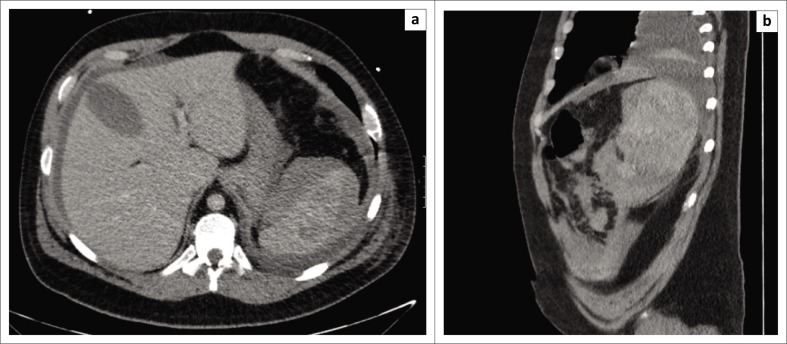
(a) Axial and (b) coronal images on day 10 post admission showing an enlarged spleen with an increased size of the subcapsular and intraparenchymal haematomas and free fluid surrounding the spleen and liver.

A 35-year-old gentleman presented to the emergency department (ED) following a motor-bicycle accident (MBA). He was known to have congenital cleidocranial dysplasia, but an otherwise unremarkable history. On clinical assessment, the patient was haemodynamically stable with significant tenderness over the left lateral chest wall and left hip. Trauma computed tomography (CT) revealed multiple posterior rib fractures on the left side with a small haemopneumothorax, multiple splenic lacerations with a subcapsular haematoma (Figure 1) and a comminuted fracture of the left femoral head with posterior dislocation. The splenic laceration was managed conservatively with serial clinical assessment, and the left femoral head fracture was repaired operatively by total hip joint replacement on day two post admission. Later during admission, serial blood tests revealed a drop in haemoglobin concentration and the patient cardiovascularly deteriorated (day 10 post admission). An urgent CT scan revealed that the spleen had increased in size with an associated subcapsular and intraparenchymal haematoma and a moderate amount of free fluid in the pelvis (Figure 2). In view of his clinical and radiological deterioration, the patient was expedited to the operating theatre and underwent an emergency laparotomy. Intra-operative findings were of 3000 ml of frank intraperitoneal blood with clots and multiple lacerations of the spleen. Splenectomy was performed and the patient went on to make an unremarkable recovery and was discharged home a week later.

**FIGURE 3:**
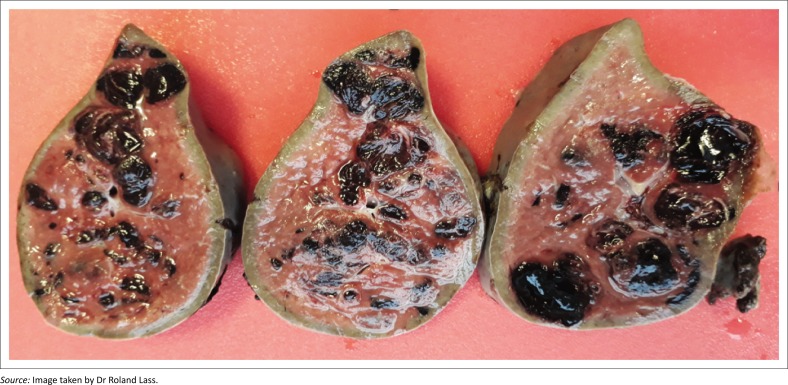
Pathology, cut specimen of the splenic parenchyma characterised by multiple blood-filled cyst-like lesions of varying sizes within the splenic parenchyma.

Histopathology noted subcapsular haemorrhage and, on sectioning, the specimen showed multiple haemorrhagic cyst-like lesions of up to 35 mm in diameter and traumatic haemorrhage (Figure 3). These cystic blood-filled lesions within the splenic parenchyma were consistent with splenic peliosis.

## Discussion

Splenic peliosis is a rare condition and often clinically silent, until found incidentally on routine scans. Splenic rupture, either spontaneous or traumatic, may result in acute presentation^5^. The etiology of splenic peliosis is often unclear, however, causal links between chronic alcoholism, corticosteroids, AIDS and the oral contraceptive pill are thought to be linked ^2^. In some cases, no predisposing link can be found^1,3^. There is a view in the literature that these represent congenital or acquired vascular malformations which manifests into cystic dilation under raised local intravascular pressure^6^. Interestingly, our case had congenital cleidocranial dysplasia. Although there has been no reported associative link in the literature between peliosis and cleidocranial dysplasia, it is plausible that these patients may be predisposed to congenital vascular malformations and hence possibly a higher incidence of peliosis.

The diagnosis of splenic peliosis relies on the radiological findings of multiple small hypo-attenuating cyst-like lesions with fluid levels mostly located around the para-follicular region of the spleen^1^. If the cysts rupture, either spontaneously or secondary to trauma, subcapsular haematoma, splenic laceration, and intra-abdominal haemorrhage will be evident. The radiological diagnosis of peliosis, or any other incidental pathology, can be very challenging in the setting of abdominal trauma, as the findings might be obscured by co-existent splenic lacerations. CT imaging interpretation may upgrade the splenic injury due to the presence of congenital haemorrhagic cysts mimicking the appearance of multiple splenic lacerations. The main CT imaging findings of peliosis are multiple well-defined or ill-defined low-density lesions, high density in presence of haemorrhage, with a variable pattern of enhancement post contrast^7^. Magnetic resonance imaging can also aid in the diagnosis of peliosis, although CT imaging is routine in trauma^1^.

The surgical management in splenic laceration with peliosis may differ from those with a normal spleen with a lower threshold for splenectomy. This is to prevent organ rupture and reduce the risk of life-threatening morbidity and mortality. Prophylactic splenectomy has been recommended in those individuals with an incidental diagnosis of splenic peliosis^6^. In the presence of trauma, surgical intervention and splenectomy is effective management, although where patients are haemodynamically stable, and resources permit, interventional radiology to perform splenic artery embolization can also be effective^8^.

## Conclusion

Isolated splenic peliosis is a rare entity, which could predispose patients to life-threatening bleeding with or without significant trauma. CT imaging plays an important role in the diagnosis and patient management, and surgical intervention is the definitive management to prevent life-threatening morbidity and mortality.

## References

[1] Davidson J, Tung K, Splenic peliosis: An unusual entity: Br J Radiology. 2010 Jun; 83; e126–e128. 10.1259/bjr/71300465 PMC3473585 20505027

[2] Begum S, Khan MR Splenic peliosis and rupture – A surgical emergency: Case report and review of the available literature. J Appl Hematol 2016;7:143–147. 10.4103/1658-5127.198508

[3] Ashbrook Daniel J, James Roger W, Philips Andrea J, Holbrook Anthony G, Agombar Andrew C – Splenic peliosis with spontaneous rupture: Report of two cases. BMC surgery. 2006;6:9 10.1186/1471-2482-6-9 16800889 PMC1523371

[4] Singh Ankur et al, Cleidocranial dysplasia with normal clavicles: A report of a novel genotype and a review of seven previous cases: Molecular syndromology. 2015;6:83–86. 10.1159/000375354; PMid:26279653 PMCid:PMC4521062 26279653 PMC4521062

[5] Singh-Ranger G, Rajarajan N, Aftab S, Splenic peliosis – A potentially fatal condition which can mimic malignancy, International seminar in surgical oncology. 2007;4:27 10.1186/1477-7800-4-27; PMid:18067667 PMCid:PMC2222050 PMC2222050 18067667

[6] Tsokos M1, Erbersdobler, Pathology of peliosis, A Forensic Sci Int. 2005 Apr 20;149(1):25–33. 10.1016/j.forsciint.2004.05.010 PMid:15734106 15734106

[7] Christop A. Karlo, Paul Stolzmann, Richard K, Katem Alkadhi, Computer tomography of the spleen: How to interpret the hypodense lesion. Insight Imaging 2013; 4;65–75. 10.1007/s13244-012-0202-z; PMid:23208585 PMCid:PMC3579987 PMC3579987 23208585

[8] Oriordan K, Blei A, Vogelzang R, Nemcek A, Abecassis M Peliosis hepatis with intrahepatic hemorrhage: Successful embolization of the hepatic artery. HPB Surg 2000;11(5):353–8. 10.1155/2000/94813; PMid:10674752 PMCid:PMC2423992 10674752 PMC2423992

